# Influence of size, shape and core–shell interface on surface plasmon resonance in Ag and Ag@MgO nanoparticle films deposited on Si/SiO*_x_*

**DOI:** 10.3762/bjnano.6.40

**Published:** 2015-02-09

**Authors:** Sergio D’Addato, Daniele Pinotti, Maria Chiara Spadaro, Guido Paolicelli, Vincenzo Grillo, Sergio Valeri, Luca Pasquali, Luca Bergamini, Stefano Corni

**Affiliations:** 1 CNR-NANO, S3, via G. Campi 213/a, 41125 Modena, Italy; 2 Dipartimento FIM, Università di Modena e Reggio Emilia, via G. Campi 213/a, 41125 Modena, Italy; 3 Centro Interdipartimentale En&TECH, Università di Modena e Reggio Emilia, via G. Campi 213/a, 41125 Modena, Italy; 4 CNR-IMEM, Parco Area delle Scienze 37/A, 43100 Parma, Italy; 5 Dipartimento di Ingegneria “Enzo Ferrari”, Università di Modena e Reggio Emilia, via Vignolese 905, 41125 Modena, Italy; 6 Department of Electricity and Electronics, Faculty of Science and Technology, UPV/EHU, 48080 Bilbao, Spain; 7 Materials Physics Center CSIC-UPV/EHU and Donostia International Physics Center DIPC, Paseo Manuel Lardizabal 4, 20018 Donostia-San Sebastian, Spain

**Keywords:** Ag, core–shell nanoparticles, electron microscopy, MgO, surface differential reflectivity, surface plasmon resonance

## Abstract

Ag and Ag@MgO core–shell nanoparticles (NPs) with a diameter of *d* = 3–10 nm were obtained by physical synthesis methods and deposited on Si with its native ultrathin oxide layer SiO*_x_* (Si/SiO*_x_*). Scanning electron microscopy and transmission electron microscopy (TEM) images of bare Ag NPs revealed the presence of small NP aggregates caused by diffusion on the surface and agglomeration. Atomic resolution TEM gave evidence of the presence of crystalline multidomains in the NPs, which were due to aggregation and multitwinning occurring during NP growth in the nanocluster source. Co-deposition of Ag NPs and Mg atoms in an oxygen atmosphere gave rise to formation of a MgO shell matrix surrounding the Ag NPs. The behaviour of the surface plasmon resonance (SPR) excitation in surface differential reflectivity (SDR) spectra with p-polarised light was investigated for bare Ag and Ag@MgO NPs. It was shown that the presence of MgO around the Ag NPs caused a red shift of the plasmon excitation, and served to preserve its existence after prolonged (five months) exposure to air, realizing the possibility of technological applications in plasmonic devices. The Ag NP and Ag@MgO NP film features in the SDR spectra could be reproduced by classical electrodynamics simulations by treating the NP-containing layer as an effective Maxwell Garnett medium. The simulations gave results in agreement with the experiments when accounting for the experimentally observed aggregation.

## Introduction

Nanoparticles (NPs) deposited on surfaces constitute a vast and important research field in material science having many applications in magnetic recording [1–2], catalysis [3], and photovoltaics [4–9]. For instance, it was found that Au NPs [5] and Ag NPs [6] deposited on thin film- and wafer-based Si solar cells can enhance their photon absorption due to the occurrence of surface plasmon resonance (SPR), which serves to scatter the incident radiation in the UV–vis region and to increase the light trapping capability. Plasmon-enhanced absorption can also be achieved by applying NPs on the rear surface of thin film solar cells, which significantly improves the performance of such devices, rendering them more cost-effective than their wafer-based counterparts [7,9]. Plasmon-enhanced performance can also be achieved in organic solar cells by incorporating Ag NPs on surface-modified transparent electrodes [8], and in LEDs by increasing their external quantum efficiency [6]. On the other hand, a number of studies on the optical properties of noble metal NPs on different surfaces have been performed [10–11], revealing the drastic dependence of the SPR oscillator strength and energy position on the NP size [12–14], geometrical shape [14–16], surface density [15–17] and the type of substrate [18]. In most of these works, NPs were realized by metal evaporation on a substrate, where islands were formed by the Vollmer–Weber growth mode [15–16]. This fundamental work can in principle be used to characterize the NPs [16] and also to obtain the optimum device performance [7,9]. Moreover, the NP synthesis can play an essential role by defining the cluster structure and the NP surface nature. Finally, the NPs can be covered with a transparent, dielectric shell, or embedded in a matrix in order to protect them from air exposure or to engineer the SPR energy position [9]. Realization of preformed, mass-selected, metal NPs by means of gas aggregation sources [19–22] allowed the systematic investigation of the NP structure and of the NP film morphology to be evaluated, relating them with “functional” properties, such as magnetic phase as a function of temperature [19]. Recently, it was also possible to co-deposit the preformed metal NPs and a flux of atoms obtained by evaporation, resulting in a core–shell structure with independently controlled core size and shell thickness [19,23–24]. This method was also used to produce a non-native oxide shell and to study the evolution of the physical properties of the NP assemblies with increasing shell thickness, owing to a configuration where NPs are embedded in a metal [19] or oxide solid matrix [23–24]. The potential of this co-deposition technique can also be exploited to study the optical properties of NP films composed of Ag cores with a transparent shell/matrix, with particular emphasis on the evolution of the shape and the energy position of the SPR. For this purpose, controlled co-deposition and systematic investigation of the NP structure, chemical composition and film morphology is crucial in order to obtain films of desired functional properties. Computation of optical spectra, which is necessary for the interpretation of the experimental data [16–18], also requires this detailed characterization information as input. In fact, electromagnetic modelling requires knowledge of the NP shape, size and arrangement of the NPs to provide results comparable with experiments.

In this work, the results of joint experimental and theoretical work on Ag and Ag@MgO core–shell NPs deposited on Si with its native, ultrathin, oxide layer, SiO*_x_* (Si/SiO*_x_*) is presented. The preformed Ag NPs, produced with a nanocluster aggregation source, were co-deposited with Mg in an O atmosphere to produce MgO shells of variable thickness. Si/SiO*_x_* was chosen for its obvious technological relevance, and MgO because of its high energy gap (*E*_g_ = 7.8 eV [25], *E*_g_ = 6 eV in ultrathin films [26]) and its efficacy in preventing metallic NPs from oxidizing in atmosphere [23]. The samples were characterized with atom force microscopy (AFM), scanning electron eicroscopy (SEM) and transmission electron microscopy (TEM) to obtain information about the NP structure and morphology, and with surface differential reflectivity (SDR) to study their optical properties in the plasmon excitation energy region. Computational simulations of the SDR spectra were performed via classical electrodynamics on the basis of the AFM, SEM and TEM experimental results. In this way it was possible to study the influence of the NP shape and size, and the MgO coverage on the plasmon resonance.

## Results and Discussion

**Figure 1:**
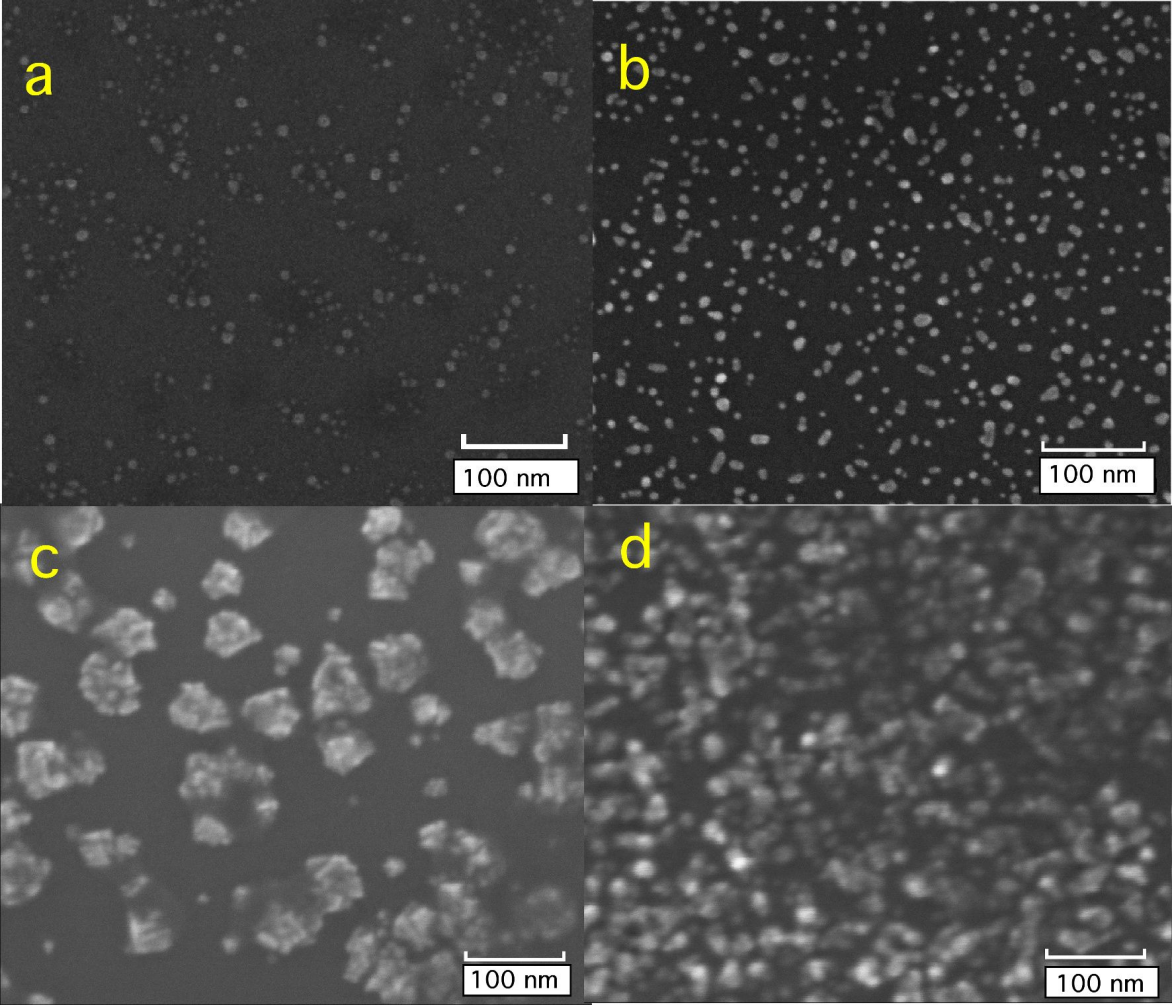
SEM images taken from (a) bare Ag NP deposited on Si/SiO*_x_* substrates with *t*_Ag_ = 0.8 nm (given in equivalent thickness), (b) bare Ag NPs, with *t*_Ag_ = 1.5 nm, (c) Ag NPs co-deposited with Mg in O atmosphere with *t*_Ag_ = 0.8 nm/*t*_MgO_ = 1.3 nm, (d) Ag NPs co-deposited with Mg in O atmosphere *t*_Ag_ = 3.3 nm/*t*_MgO_ = 4.8 nm.

Preformed Ag NPs with and without Mg were deposited on Si/SiO*_x_* substrates in O_2_ atmosphere in order to form UV–vis transparent oxide shells around the Ag nanocluster cores and to investigate their effect on the morphological and optical properties. X-ray photoelectron spectroscopy analysis after deposition in vacuum revealed that the Ag 3d core level lineshape was unaffected by the co-deposition procedure and the Mg 1s spectra did not show any significant plasmon loss, as was previously observed in Ni@MgO and FePt@MgO core–shell NPs prepared with the same procedure [22–23]. This confirmed that while the Ag NPs remained in a metallic state, the Mg was mostly oxidized. Figure 1 reports typical SEM images from four different films: (a) bare NPs with a nominal thickness of *t*_Ag_ = 0.8 nm, (b) bare NPs with *t*_Ag_ = 1.5 nm, (c) Ag NPs co-deposited with MgO (*t*_Ag_ = 0.8 nm/*t*_MgO_ = 1.3 nm), and (d) Ag co-deposited with MgO (*t*_Ag_ = 3.3 nm/*t*_MgO_ = 4.8 nm). The quantity of MgO deposited was chosen in order to maintain an approximately constant Ag/MgO ratio (given by the ratio between the two nominal thickness values, *t*_Ag_/*t*_MgO_ = 1.4 or *t*_Ag_/*t*_MgO_ =1.6).

The Ag NPs are clearly visible in the SEM image in Figure 1a, and the grain analysis (performed by fitting the diameter distribution with a log–normal function) gave an average lateral size of <*d*> = 6.5 ± 1.9 nm [27]. The vertical height distribution was also measured with AFM, giving an average value of <*h*> = 3.2 ± 0.1 nm (see Supporting Information File 1). The average aspect ratio (AR) of the NPs, defined as AR = <*d*>/<*h*>, was found to be AR = 2.0 ± 0.6. The high value of the estimated AR originates from deformation of a single NP, caused by interaction with the substrate and by the presence of small agglomerates during NP aggregation. With increasing values of *t*_Ag_ (i.e., when the number of deposited Au NPs is increased), the presence of NPs with elongated or irregular shapes (*d* ≈ 10–30 nm, Figure 1b) can be observed from SEM with a higher density of NPs, as expected. This effect can be ascribed to diffusion of the deposited Ag nanoclusters on the substrate and formation of aggregates.

**Figure 2:**
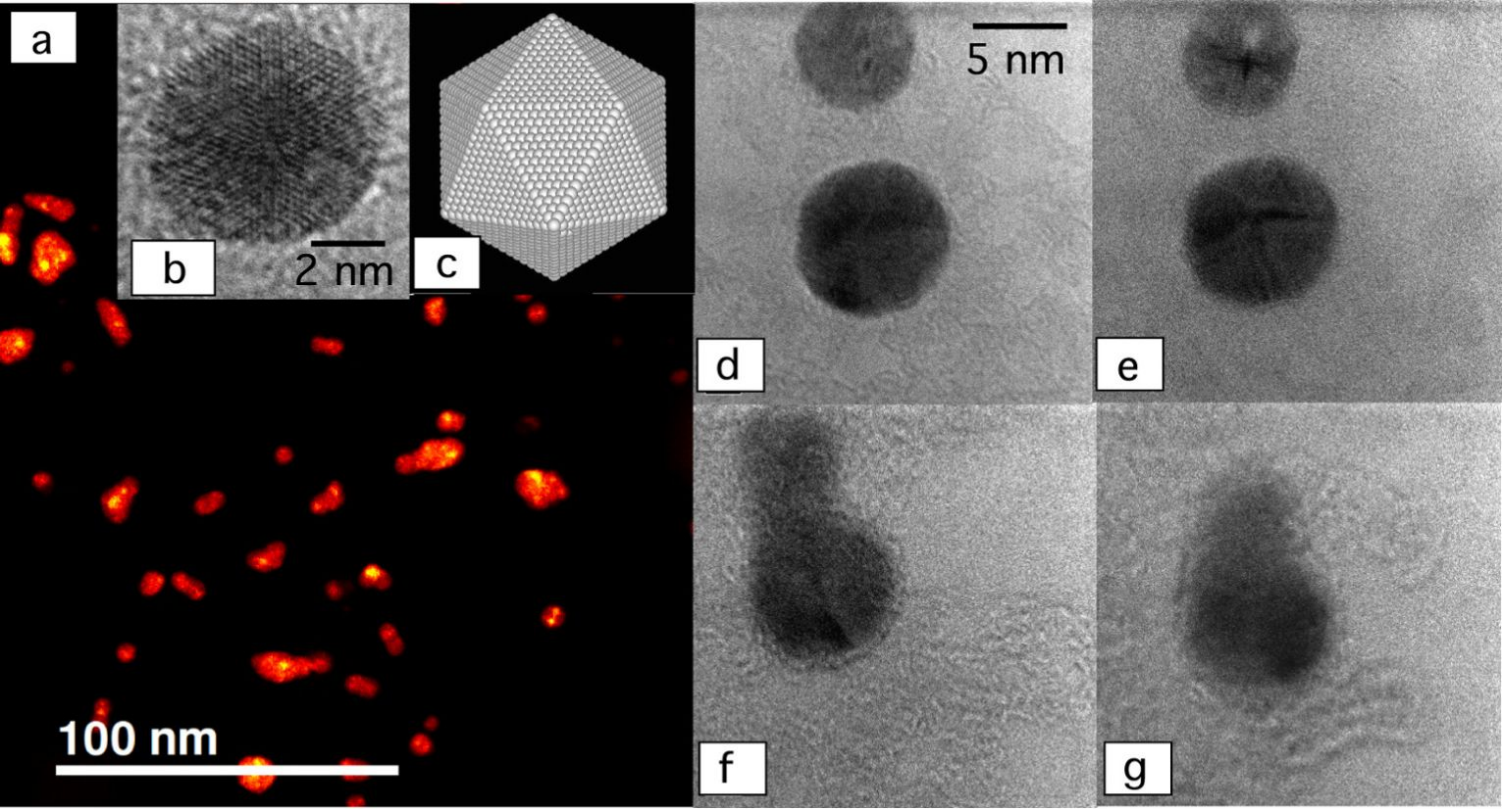
(a) STEM–HAADF image of Ag NPs, (b) atomically resolved TEM image of a single NP, revealing crystallite multitwinning corresponding to a McKay icosahedral geometry, (c) atomistic model of a NP with a McKay icosahedral geometry, and (d–g) sequence of TEM images taken at time intervals *t ≈* 60 s, showing diffusion and aggregation of two Ag NPs.

SEM images of Ag@MgO NP films show a drastically different morphology. In Figure 1c the presence of sparse agglomeration on the Si/SiO*_x_* substrate with variable size can be observed. Some single NPs can be also distinguished. Within the agglomerates (which can be as large as *d* = 80 nm) a granular structure can be observed due to the presence of the original Ag NPs. As observed on Ni@MgO [22] and FePt@MgO [23], MgO preferentially grows around the NPs. This is due to a much higher sticking coefficient of the metal compared to the inert Si/SiO*_x_* surface, and the MgO tends to form a matrix embedding the original particles. The same situation holds for the case of Figure 1d, although the higher quantity of deposited Ag NPs gives rise to more diffused agglomerates, covering most of the substrate area. Aggregation of the NPs was also observed with scanning TEM–high angle annular dark field (STEM–HAADF) (Figure 2a) and TEM (see also Supporting Information File 1). Areas with different crystal domains were observed and are evidently caused by crystal twinning, which occurs during the NP growth process [28] or by formation of NP agglomerates with different crystal orientations after deposition and diffusion on the substrate [29–31]. Figure 2b shows an atomic resolution TEM image of a single NP. The image corresponds to a McKay icosahedral geometry, where the icosahedron is assembled from single crystal tetrahedra with (111) faces [28,32] (see Figure 2c). This type of structure, as previously observed in other fcc metal NPs [22–23,28,33], can be ascribed to the dynamics of NP growth. In particular, it was found that formation of icosahedra is favoured at fast quenching rates for fcc metal NPs [33–35].

Interestingly, diffusion and agglomeration of the as-deposited Ag NPs could be observed in situ during TEM experiments, as reported in Figure 2d–g, where a sequence of TEM images taken at time intervals of approximately 60 s show two NPs approaching and eventually forming a dimer. Diffusion in this case was probably influenced by sample heating caused by the microscope electron beam.

**Figure 3:**
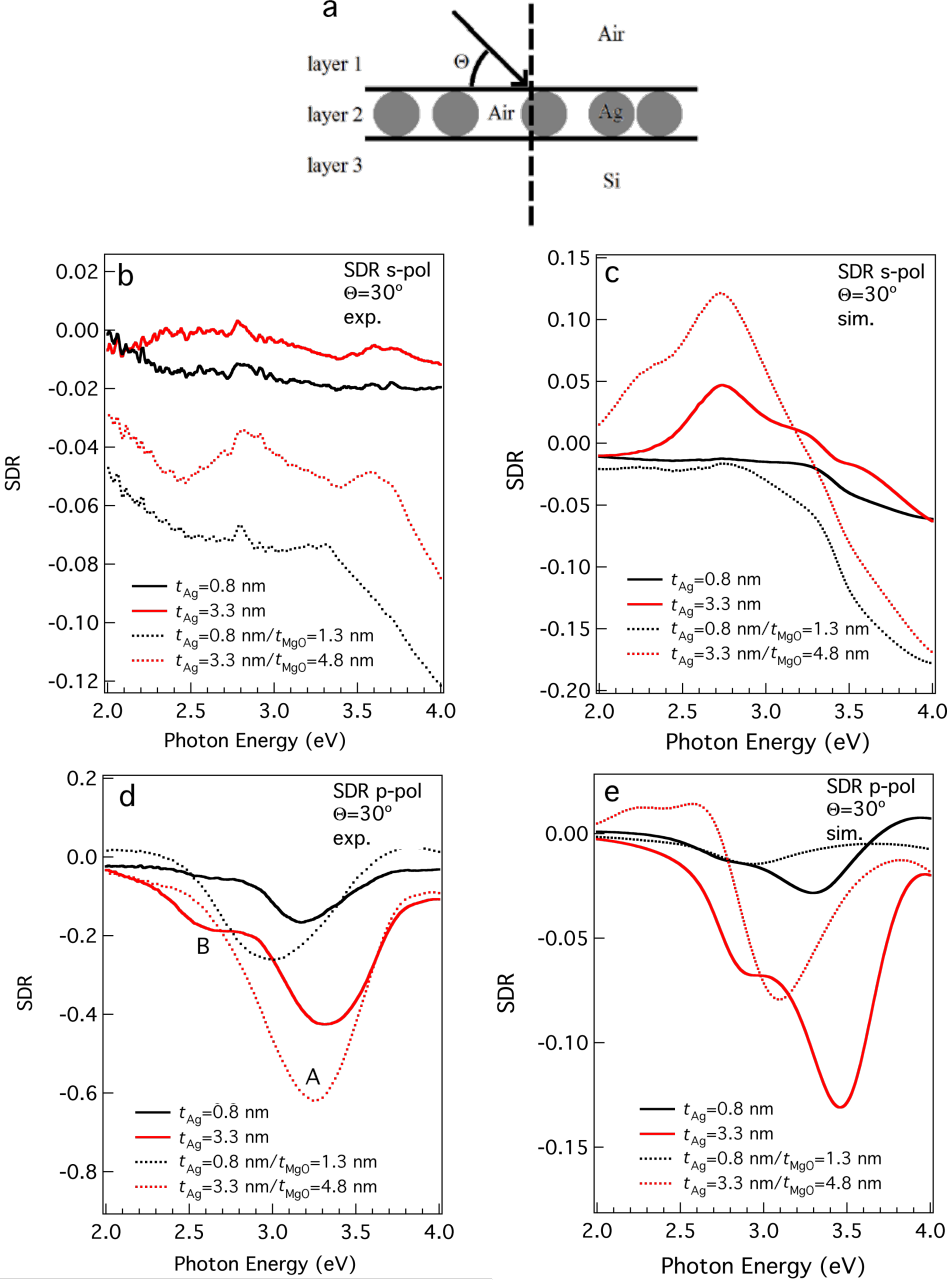
(a) Sketch of the geometry for the SDR experiments showing the incidence angle, Θ, and the system used for the simulation of the Ag NP films. (b) Experimental SDR spectra obtained under s-polarisation geometry from bare (continuous line) and MgO-covered (dotted line) Ag NPs deposited on Si/SiO*_x_*, with incidence angle Θ = 30°. (c) Simulated SDR spectra for s-polarised incident radiation with Θ = 30°. Results are shown for a nominal thickness of the NP layer *t*_Ag_ = 0.8 nm (black curves) and *t*_Ag_ = 3.3 nm. (d) Corresponding experimental SDR curves obtained under p-polarisation geometry. (e) Simulation results under p-polarisation illumination.

The reflectivity spectra were taken as a function of the incidence angle, Θ, between Θ = 15° and Θ = 60°. The incidence angle is defined here with respect to the substrate surface (see Figure 3a). As such, small values of Θ are found at almost grazing incidence, whereas values approaching 90° indicate almost normal incidence (radiation perpendicular to the substrate surface). The experimental surface differential reflectivity (SDR, SDR = (*R*_Ag_ / *R*_Si_) − 1) was obtained by measuring the reflectivity spectrum of the Ag NP films deposited on Si/SiO*_x_*, *R*_Ag_, and the reflectivity spectrum of the Si/SiO*_x_* substrate, *R*_Si_. The SDR spectra were obtained from bare Ag NP (*t*_Ag_ = 0.8 nm, *t*_Ag_ = 3.3 nm) and on Ag NP/MgO (*t*_Ag_ = 0.8 nm/*t*_MgO_ = 1.3 nm, *t*_Ag_ = 3.3 nm/*t*_MgO_ = 4.8 nm) films deposited on Si/SiO*_x_* substrates, as discussed in the experimental section. Figure 3 shows SDR spectra taken at angle of incidence of Θ = 30°, with incident radiation of s- and p-polarisation, together with classical electrodynamics simulations. For s-polarised incident radiation, it can be readily observed that SDR data from bare NPs do not reveal any significant features in the photon energy region between 2 and 4 eV (Figure 3b). In the case of MgO-covered NPs, the s-polarised spectra present two weak structures superimposed on a decreasing slope.

The case for SDR spectra taken with p-polarisation excitation is very different (Figure 3d). In this case, a well-defined minimum (labelled A) is observed for all thicknesses and is located at energies between *hv* = 3.0 eV and *hv* = 3.3 eV. This minimum is much more intense (as deep as SDR = −0.6) than for the structures observed under s-polarisation, with corresponding intensity oscillations of about 0.02. A shoulder is also observed in the spectra obtained from bare NPs, positioned at *hv* = 2.6 eV (feature B in Figure 3d). The minimum (A) can be clearly assigned to excitation of the surface plasmon, however, its exact position depends on the amount of deposited Ag NPs and also on the presence of MgO. Also, the shoulder B is not present when NPs are covered with MgO.

**Figure 4:**
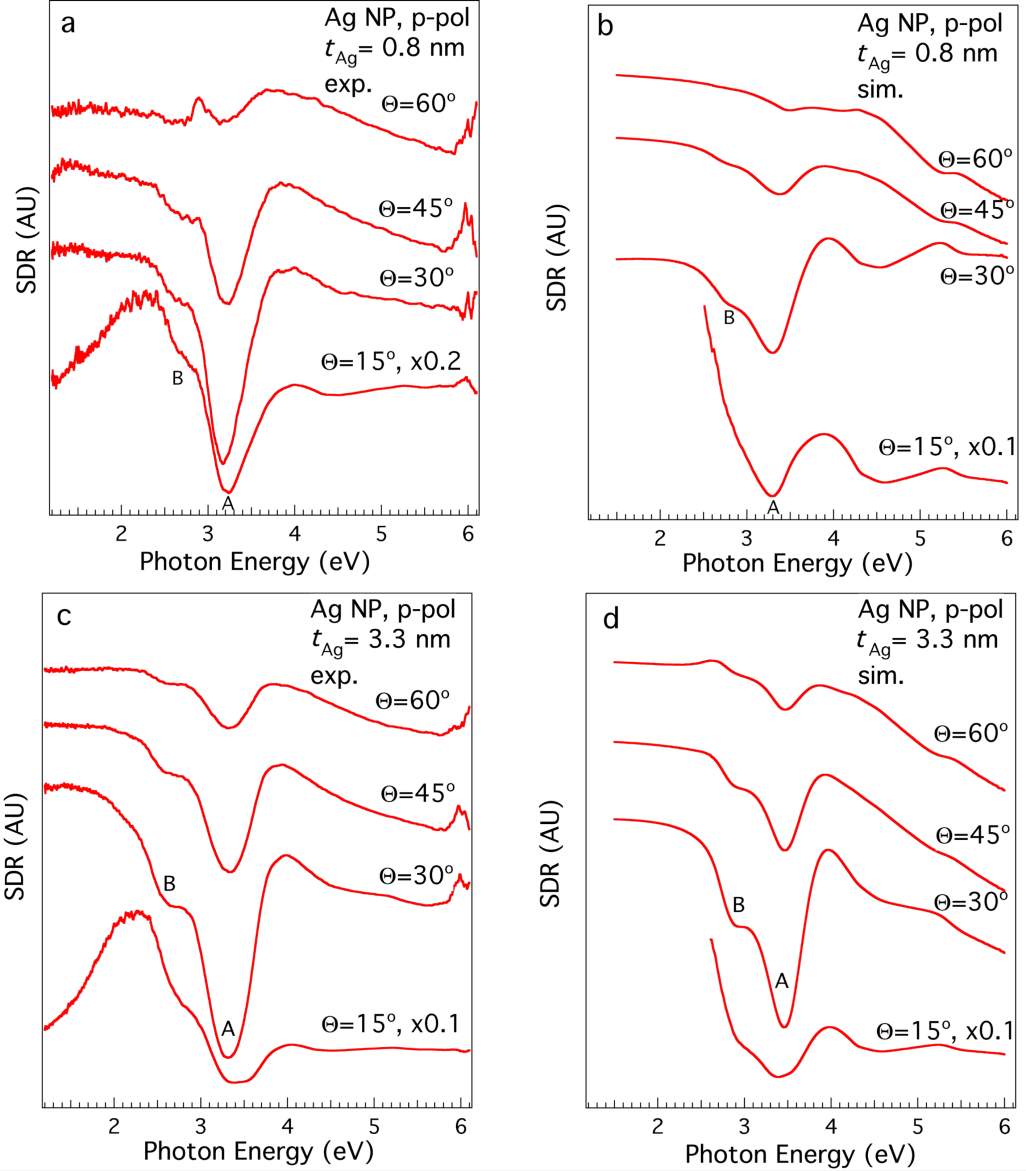
(a) SDR spectra of bare Ag NPs deposited on Si/SiO*_x_*, *t*_Ag_ = 0.8 nm, taken under p-polarisation at different values of Θ. (b) Simulated SDR spectra for a 0*.*8 nm nominal thickness of the AgNP layer (*t*_Ag_). The calculations were performed at varying incidence angles according to the experiment from (bottom) almost grazing incidence to (top) almost normal incidence. The spectra refer to the deposition of bare Ag NPs. (c) Experimental p-polarised SDR data for *t*_Ag_ = 3*.*3 nm. (d) Simulated SDR spectra for *t*_Ag_ = 3.3 nm.

The spectra of Figure 3d show evidence of a blue shift of feature A with increasing values of *t*_Ag_, and also of a red shift when the same amount of Ag NPs is co-deposited with MgO. In order to understand the behaviour of the SDR spectra, simulations were performed following the method explained in the experimental and computational sections. It was assumed that the layer containing the NPs was of 5 nm thickness in order to account for the NP height distribution measured by AFM. Since the effective dielectric function of the medium is not directly sensitive to the NP size, but rather to the overall volume fraction and NP shape, an accurate targeting of the experimental NP height is not needed, and the chosen value of 5 nm is a good compromise to be used for both *t*_Ag_ = 0*.*8 nm and *t*_Ag_ = 3.3 nm. Firstly, we considered the system composed of bare Ag NPs with a nominal thickness of *t*_Ag_ =0*.*8 nm (Figure 3e, continuous black curve). Good agreement between the experimental and theoretical outcomes were achieved assuming that the NPs occupy approximately 1*.*6% of the layer volume (*f* = 0*.*016, where *f* = *V*_AgNPs_/*V*_layer2_), which is a reasonable value on the basis of the particle distribution revealed by SEM (see Figure 1a). Moreover, the best match with experimental data was found when the NP ensemble is assumed to be a mixture of spherical NPs (with diameter of 4 nm) and prolate spheroidal NPs (with two minor semiaxes of 4 nm and a major 6*.*5 nm long semiaxis) in a ratio of 50/50. Nanospheroids are representative of the Ag NP aggregates originated by the coalescence process, in accordance with the experimental observations (see Figure 2). The plasmon resonance linked to the nanospheres and the minor axes of the nanospheroids causes the deep recess (minimum) around 3*.*5 eV, which slightly blue-shifted with respect to the film thickness ratio (feature A in Figure 3d and Figure 4a). The plasmon resonance along the major semiaxis of the prolate nanospheroids is the cause of the shoulder around 3 eV (labeled as B in Figure 3d and Figure 4a). The exact positions of the recess and the shoulder are also sensitive to the surrounding environment of the NPs. Since the SDR measurements were carried out in atmosphere, it is reasonable to assume that a thin film of water of a few Å covers the surface and the NPs. Taking into account that this water layer is in direct contact with the NPs, we assumed the extreme case of ε_M_ = 1.77 (i.e., the optical constant of water) for the dielectric function of the embedding medium in the Maxwell Garnett dielectric function, where layer 1 was always assumed to be air (see Figure 3a). The other extreme, that is, the case of particles with no water layer (i.e., fully in air), was also explored and with the results presented in Supporting Information File 1, which reveals very similar shapes of the SDR curves. Therefore, the choice of this embedding medium dielectric constant, provided it is in the reasonable range of 1 to 1.77, is not decisive to reproduce the experimental trends (see Supporting Information File 1).

Simulations were also performed for the sample with a silver nominal thickness of *t*_Ag_ = 3*.*3 nm, shown in Figure 3c,e and Figure 4d. In this case, the best *f* was found to be 0*.*08, quantitatively in accordance with the increased nominal thickness. The nanosphere/nanospheroid ratio that provides the best reproduction of the experimental trends is different: in this case, the best nanosphere fraction is around 10%. The dominance of nanospheroids is in qualitative accordance with the experimentally observed increase of agglomeration when the concentration of Ag NPs is increased.

The SDR spectra of the sample formed by deposition of Ag NPs covered in MgO (Ag@MgO NPs) were also simulated. From the experimental spectra (Figure 3d) it is noticeable that the shoulder B disappears. This can be ascribed to the fact that the MgO coating prevents the NPs from agglomerating, thus resulting in a quenched production of dimers or other smaller aggregates. Indeed, for both thicknesses, the simulations provide a good match with the experiments when the quantity of nanospheroids is less than that of the nanospheres (compare Figure 3e with Figure 3c). For *t*_Ag_ = 0*.*8 nm, the best spectrum reproduction was found for a nanosphere/nanospheroid ratio of 70:30 (*f* = 0.016 was used, as for the uncovered NPs). In the case of *t*_Ag_ = 3*.*3 nm, the ratio was 60:40 (again *f =* 0.08, as for the uncovered NPs). The red shift of the plasmon feature is also well-reproduced and appears to originate by assuming MgO as the matrix medium embedding the NPs.

To investigate the behaviour of the plasmon feature in SDR, spectra were taken under p-polarisation at different values of Θ (Figure 4). As it can be seen in Figure 4, when passing from Θ = 60° (corresponding to almost normal incidence) to Θ = 30° (grazing incidence), there is a sharpening of the plasmon feature A, as well as an increasingly defined, low-energy shoulder B. Further decrease of the incidence angle seems also to produce a further enhancement in the intensity of the minimum A.

The simulated data show an overall good agreement with the experimentally observed behaviour for both thicknesses, confirming the quality of the proposed electromagnetic model and the interpretation of the results. Note that the curves for the grazing angle (Θ = 15°) show a divergence for small photon energies (< 2.5 eV). This can be attributed to numerical inaccuracy rather than to a physical effect: since the reflectivity spectrum for this grazing angle approaches zero in both cases, the ratio (*R*_sub+AgNPs_ − *R*_sub_) / *R*_sub_ defining the SDR can give rise to numerical instability.

**Figure 5:**
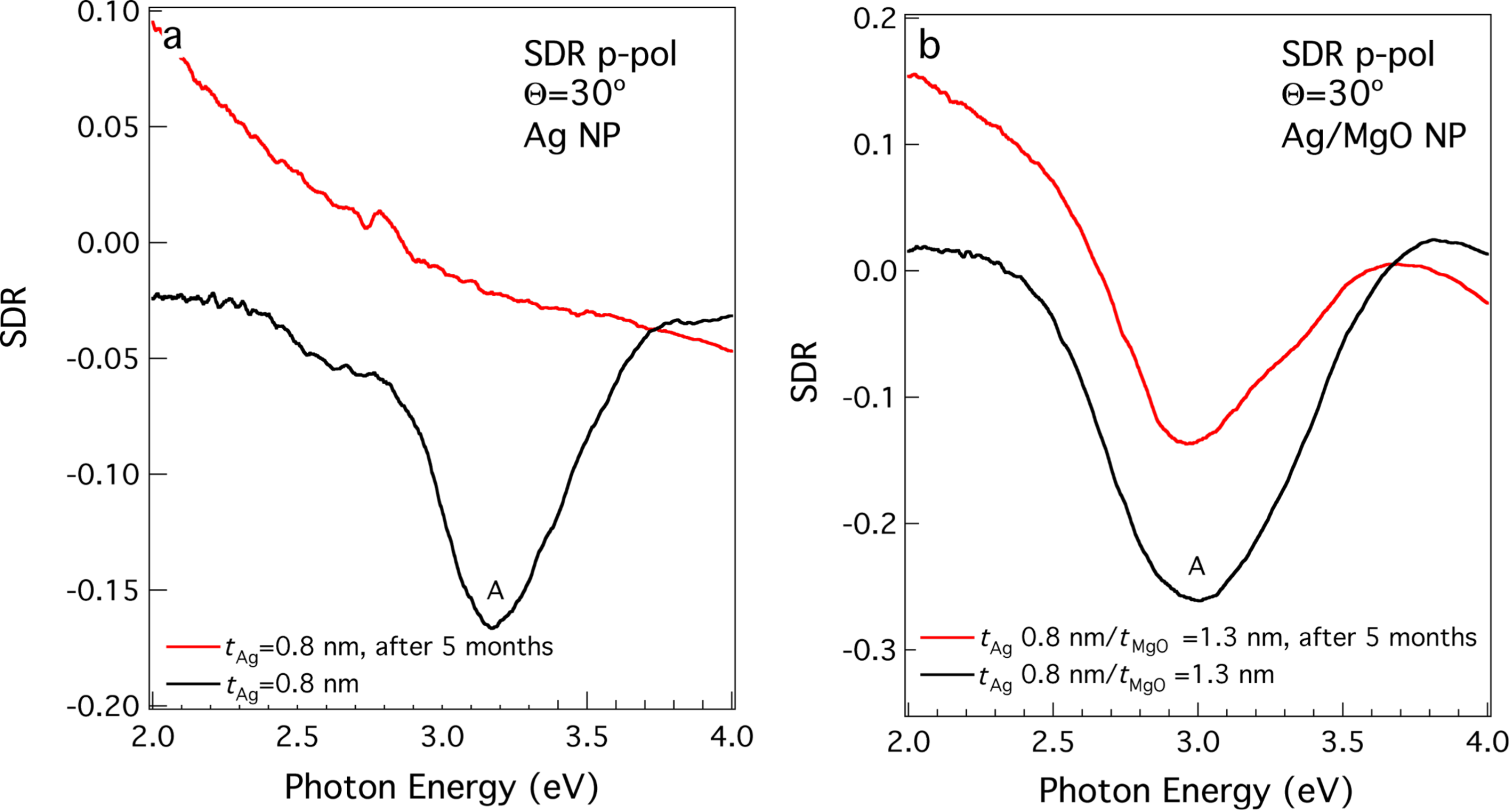
(a) SDR spectra taken under p-polarisation excitation at Θ = 30° on Ag NPs deposited on Si/SiO*_x_* after few days (black curve) and after five months of exposure to air. (b) Same as (a) but with Ag NPs co-deposited with MgO.

Finally, the effect of the MgO layer as a protective ultrathin coating for the optical properties of Ag NPs was evaluated by taking SDR spectra after a prolonged time after the preparation of the samples. Figure 5 shows SDR data from Ag NPs and Ag@MgO NPs few days after deposition and after exposure to air for five months. It can be seen that the plasmon feature has completely disappeared in the case of bare Ag NPs, while it is preserved when Ag NPs are co-deposited with MgO, even in the limit of ultrathin (*t*_MgO_ = 1.3 nm) protective layers. This result is relevant for possible applications in photovoltaics and in other fields where Ag NPs are used for their plasmonic properties.

## Conclusion

The results of an investigation on the morphology, structure and optical properties of bare Ag and Ag NPs co-deposited with MgO on Si/SiO*_x_* have been presented. The Ag NPs, generated with a gas aggregation source, diffused on the substrate and formed small aggregates, increasing in size with increasing quantity of deposited material. TEM images showed the presence of multidomains, either due to the aggregation of NPs with different crystal orientation (one of such aggregation events could be followed by TEM images taken at different times) or to multitwinning occurring during the NP formation in the source. When co-deposited with Mg in O atmosphere, SEM images showed grains, which were assigned to formation of MgO shells around the original Ag NPs. SDR spectra taken under p-polarisation excitation exhibited a plasmon feature (with a deep minimum) at a photon energy between 3 and 3.5 eV where the intensity of this peak decreases at higher incidence angles. This plasmon feature was not observed with SDR under s-polarisation excitation, that is, it could only be excited when the electric field of the radiation was normal to the sample surface. This behaviour was expected for the continuous film due to the given NP size and interparticle distance relative to the radiation wavelength. SDR calculations were performed based on the Fresnel equations and a three-layer model with a film (corresponding to the NPs) where the Maxwell Garnett formula was assumed for the dielectric function given the ellipsoidal inclusions with different phases. The NPs were simulated by a mixture of spheres and ellipsoids to take into account the effect of aggregation and the interaction of the substrate experimentally observed. The simulations were in good agreement with the experimental results, reproducing the blue shift of the plasmon observed at increasing incidence angle, and the presence of a second feature at lower photon energies in the spectra of bare particles, which was assigned to the elongated shapes of the small NP aggregate and the absence of plasmon resonance in SDR at s-polarisation was also reproduced. When covered with MgO, the SDR exhibited a red shift due to the Ag–MgO interface and disappearance of the second feature. This is assumed to be due to the fact that NP diffusion and aggregation is hampered by the MgO shell/matrix. The stability of optical properties of Ag@MgO NP films after prolonged exposure to air was also demonstrated, providing the possibility of employing MgO as a transparent coating in plasmonic devices.

### Experimental and Computational Details

Ag NP and Ag@MgO NP films were prepared in an experimental system with three interconnected vacuum chambers described elsewhere in detail [27–28]. The first chamber was equipped with a gas aggregation NP source, composed of a magnetron (NC200U, Oxford Applied Research) and a quadrupole mass filter (QMF). Ag NPs were deposited in vacuum or co-deposited with Mg atoms obtained by thermal evaporation in O_2_ atmosphere, with a similar procedure used to obtain Ni@MgO and FePt@MgO NP [22–23]. The deposition rate of the different materials was monitored with a quartz microbalance, and the film chemical composition was analysed with XPS. For the experiments reported in this work, the samples were produced with a NP beam generated with magnetron discharge power *P* ≈ 35 W, and Ar flow value between 40 sccm and 60 sccm. In these conditions we could obtain Ag NP with a linear size distribution between 3 and 10 nm, as measured by the QMF and directly verified by the SEM and TEM images. The size distribution of the deposited particles was always checked ex situ with SEM and with TEM [22–24,27–28]. The quantity of deposited Ag NPs and the resulting MgO (see the Results and Discussion section) are given in this work in terms of nominal thickness of an equivalent continuous film with the same density as bulk fcc Ag and rock salt MgO. The O_2_ partial pressure and Mg deposition rate were adjusted in order to obtain Ag NPs embedded in MgO. A typical O_2_ pressure value was *P*_O2_ = 2 × 10^−7^ mbar, while the Ag NP deposition rate varied between 0.1 and 0.20 nm/min. The MgO deposition rate varied between 0.3 and 0.8 nm/min. Inert substrates were used during experiments, in particular: (i) Si with an ultrathin film of native oxide SiO*_x_* (Si/SiO_x_) for SEM, XPS, and optical measurements, and (ii) Carbon-coated copper grids for TEM. p-doped Si wafers with native oxide SiO*_x_* (Si/SiO*_x_* substrates) were rinsed in methanol and introduced in the deposition chamber. A value *t*_SiO_*_x_* ≈ 0.5 nm for the oxide layer thickness was estimated from XPS analysis of the Si 2p core level peaks.

As previously reported in the works on different NP films [22–24], SEM images were taken with a dual beam system (FEI Strata DB235M), while TEM and STEM–HAADF mode images were collected with a JEOL JEM-2010 (200 keV) operating with a LaB_6_ source, and a JEOL JEM-2200FS working at 200 KeV and equipped with Schottky field emission gun.

Optical reflectivity experiments were performed in air using linearly polarised, s- and p-polarised radiation. The experimental system for these measurements was equipped with: an Ocean Optics DH-20000-BAL light source, emitting radiation with wavelength in a range between 200–1050 nm; polarisers; and an Ocean Optics HR4000CG-UV-NIR grating monochromator, equipped with CCD detectors.

Simulations of the optical properties of the experimental system were carried out by using classical law of geometrical optics. In particular, the Fresnel equations for reflection, refraction and absorption by a dissipative multilayer [36–37] with a plane wave at arbitrary incidence were used. These formulations were implemented in the framework of a custom-written Fortran code. The optical properties of the involved media were inferred by employing either dispersive, dissipative dielectric functions (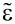) or dielectric constant (ε). In particular, the dielectric function of the substrate (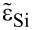) and of the MgO covering layer (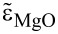) were obtained by a cubic spline interpolation of the experimental data for the crystalline silicon and MgO, respectively, by Palik [40]. The optical behaviour of the silver comprising the NPs is provided by the fit of the Lynch & Hunter silver data [38] by Blaber, et al. [39]. Since the size of the involved NPs is of a few nanometers, we additionally corrected the fit with a mean free path correction used for spheres [40]. The medium in contact with the incident radiation is assumed to be vacuum (ε_1_ = 1), whereas, over the range of frequencies in the selected calculations, the dielectric constant for water ε_H2O_ = 1*.*77 is taken [41] (see the Results and Discussion section). In particular, the realized samples were modelled as three-layer systems (see Figure 3a). Layer 1, which receives the incident light, is assumed as air (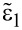 = 1). The substrate (layer 3) is assumed to be bulk silicon (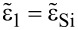). In fact, it was verified that the optical behaviour of the substrate is determined by Si only, that is, that the contribution of a possible top Si oxide layer is negligible (see Supporting Information File 1). This hypothesis is reinforced by the low value of the thickness of the oxide layer estimated by XPS. The silver NPs are modelled by means of a layer (layer 2) whose optical properties are inferred from effective medium theory (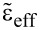). Namely, the layer is assumed to have a Maxwell Garnett dielectric function (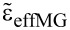) [42].

[1]
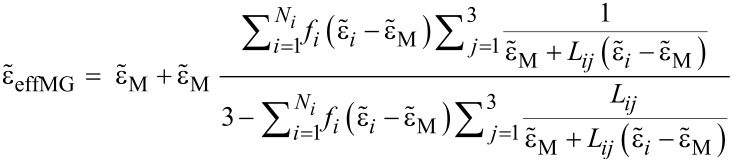


This dielectric function was used to mimic the effect of inclusions embedded in a matrix: it does not account for their size distribution but only for their volume fraction, *f*. However, the general Maxwell Garnett theory can also take into account inclusions of different shapes (or aspect ratios), orientation and material composition, referred to as phases. In the present calculations the Maxwell Garnett formula for ellipsoidal inclusions with different phases was employed as given by [42] Equation 1, where 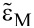 is the dielectric function of the embedding matrix (1.77 for the results in the main text, 1 for the results in Supporting Information File 1, Figure S1), 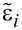 is the dielectric function of the *i*th type of inclusions (*i* phase), *f_i_* is the volume fraction occupied by the inclusions of the *i*th type, *L_ij_* are the depolarisation factors of the *i*th type of inclusion, and the index *i* = 1*, . . . , N_i_* spans over all phases present in the mixture, whereas the index *j* = 1*,* 2*,* 3 corresponds to *x*, *y*, and *z* Cartesian coordinates. Equation 1 reduces to the well-known Maxwell Garnett formula with spherical inclusions when only nanospheres of the same material are considered (i.e., when *i* = 1 and *L*_11_ = *L*_12_ = *L*_13_ = 1*/*3) [10,43–44]:

[2]
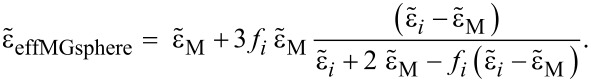


## Supporting Information

File 1 Additional AFM, TEM, SDR and simulation data.
Additional AFM and TEM images of bare Ag NP, simulations of SDR spectra of bare Ag NP with air as an effective 2nd layer medium, and simulations of reflectivity from Si, SiO_2_/Si and SiO/Si compared with experimental data.
