# Supplementary material for: Influence of size, shape and core–shell interface on surface plasmon resonance in Ag and Ag@MgO nanoparticle films deposited on Si/SiOx
Source: Beilstein J Nanotechnol. 2015 Feb 9;6:404–13. doi: 10.3762/bjnano.6.40 (PMC4362337; doi:10.3762/bjnano.6.40)
Supplement: File 1 — Additional AFM, TEM, SDR and simulation data. Additional AFM and TEM images of bare Ag NP, simulations of SDR spectra of bare Ag NP with air as an effective 2nd layer medium, and simulations of reflectivity from Si, SiO2/Si and SiO/Si compared with experimental data. [file Beilstein_J_Nanotechnol-06-404-s001.pdf]

Influence of size, shape and core–shell interface on surface plasmon resonance in Ag and Ag@MgO nanoparticle films deposited on Si/SiO$_x$

Sergio D'Addato*[1,2,3,§], Daniele Pinotti[2], Maria Chiara Spadaro[1,2], Guido Paolicelli[1], Vincenzo Grillo[1,4], Sergio Valeri[1,2], Luca Pasquali[5], Luca Bergamini[1,2,6,7], and Stefano Corni[1]

Address: [1]CNR-NANO, S3, via G. Campi 213/a, 41125 Modena, Italy, [2]Dipartimento FIM, Università di Modena e Reggio Emilia, via G. Campi 213/a, 41125 Modena, Italy, [3]Centro Interdipartimentale En&TECH, Università di Modena e Reggio Emilia, via G. Campi 213/a, 41125 Modena, Italy, [4]CNR-IMEM, Parco Area delle Scienze 37/A, 43100 Parma, Italy, [5]Dipartimento di Ingegneria "Enzo Ferrari", Università di Modena e Reggio Emilia, via Vignolese 905, 41125 Modena, Italy, [6]Department of Electricity and Electronics, Faculty of Science and Technology, UPV/EHU, 48080 Bilbao, Spain and [7]Materials Physics Center CSIC-UPV/EHU and Donostia International Physics Center DIPC, Paseo Manuel Lardizabal 4, 20018 Donostia-San Sebastian, Spain

E-mail: Sergio D'Addato* - sergio.daddato@unimore.it

[*] Corresponding Author

[§]Tel.: +39-059-2055254, Fax: +39-059-2055235

Additional AFM, TEM, SDR and simulation data.

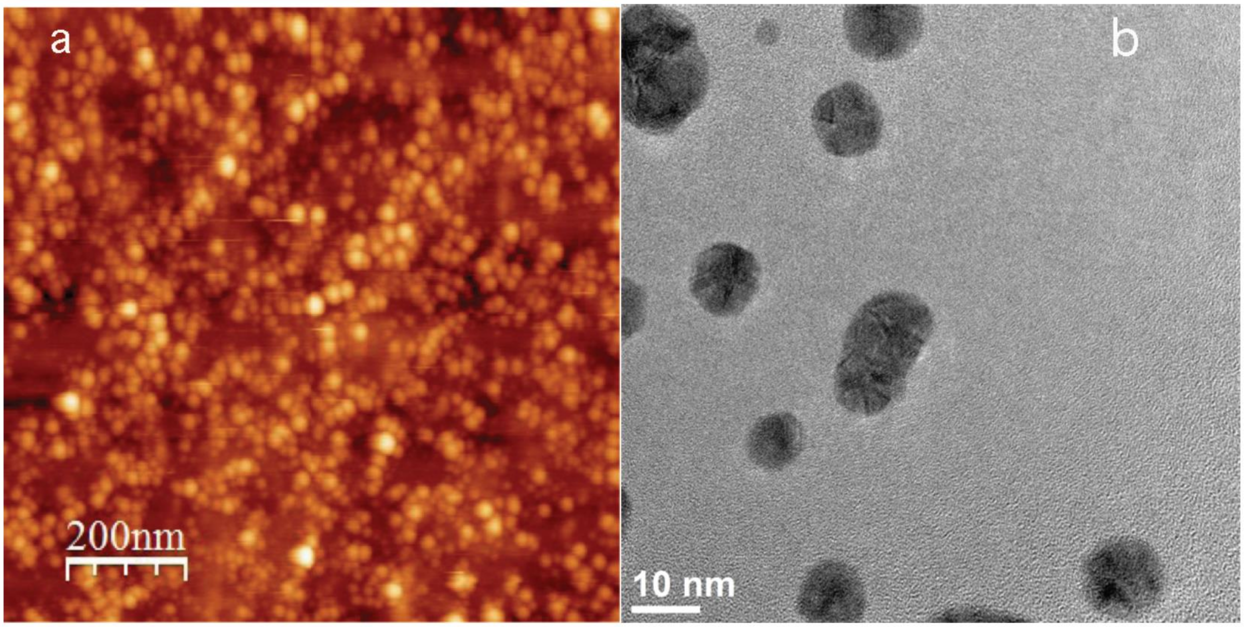

Figure S1: (a) AFM image of Ag NP film deposited on a Si/SiO*x* substrate with nominal thickness t_{Ag} = 0.8 nm. The sample used in the AFM experiment was the same as the one imaged with SEM in Figure 1a of the manuscript. The Ag NPs imaged by AFM have an apparent size much bigger than in SEM because of the probe limits the lateral resolution determined by the radius of the AFM tip, which is estimated to be $R_t \approx 15$ nm [1]. Nevertheless, AFM images were analysed in order to obtain information about the NP vertical height distribution. The obtained average height was $\langle h \rangle = 3.2 \pm 0.1$ nm. (b) TEM images of Ag NPs, showing agglomeration of two or three NPs. The NP internal structure is due to multitwinning occurring during the NP growth.

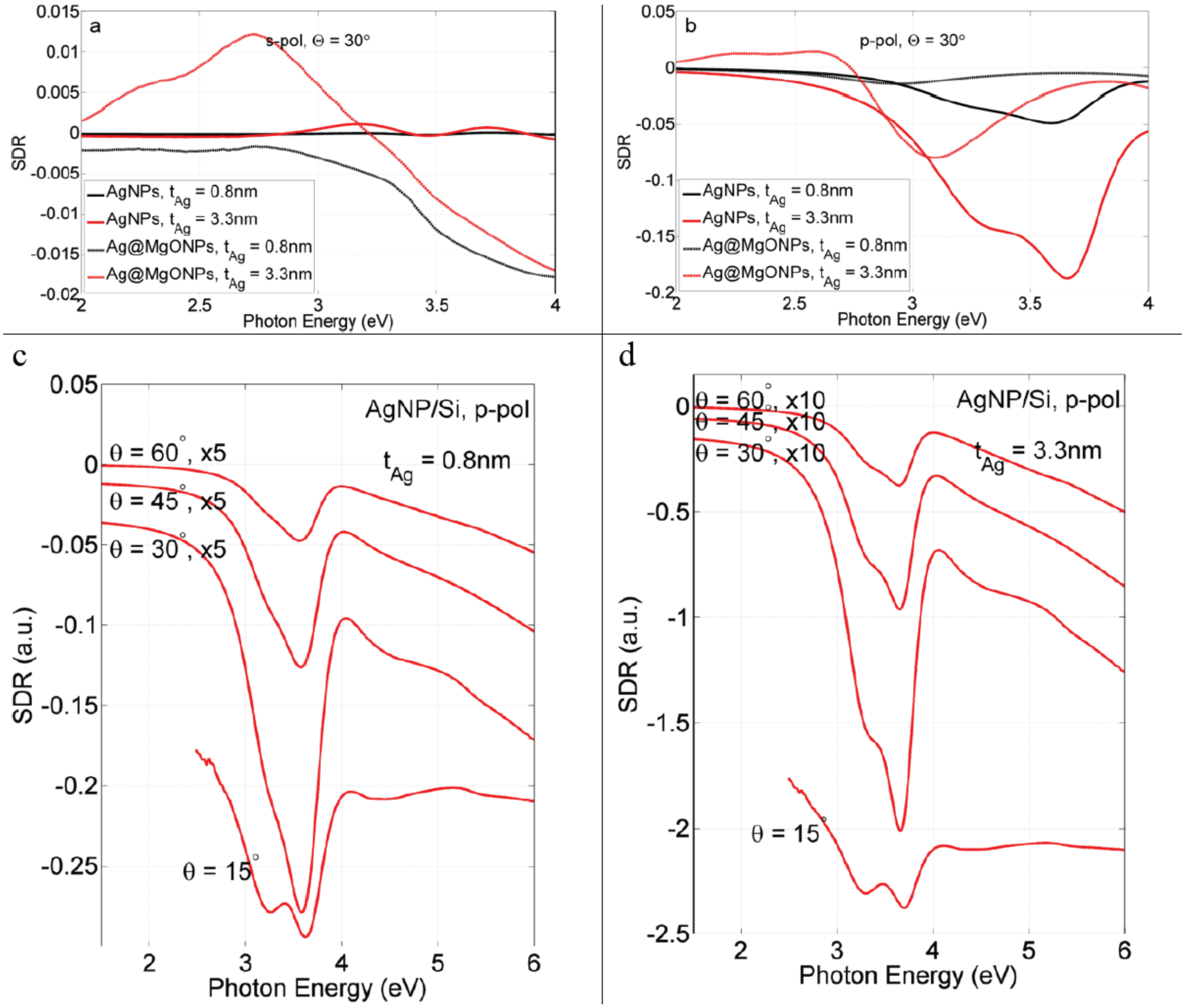

Figure S2: (a,b) Simulated SDR spectra for s-polarized and p-polarized incident radiation, with Θ = 30º. (c,d) Simulated SDR spectra for a 0.8 nm and 3.3 nm nominal thickness of the AgNP layer (t_{Ag}). These calculations are performed at varying incidence angle according to the experimental observations from (bottom) almost grazing incidence to (top) almost normal incidence. All the panels in this figure refer to an effective medium dielectric function where the presence of a water layer is discarded (i.e., NP in air only). These plots should be compared with the corresponding ones in the main text where a wetting water layer is considered. The main difference is a general blue shift of the plasmon peaks in air. Moreover, the s-polarized spectra in panel (a) are also less intense. Finally, the Θ = 150 spectrum in panel (c) has different features both of the plasmon peak and of the tail at higher energy. In this case, the corresponding experimental data in the main text seem intermediate between the "water-layer" and "no water-layer" situation as expected.

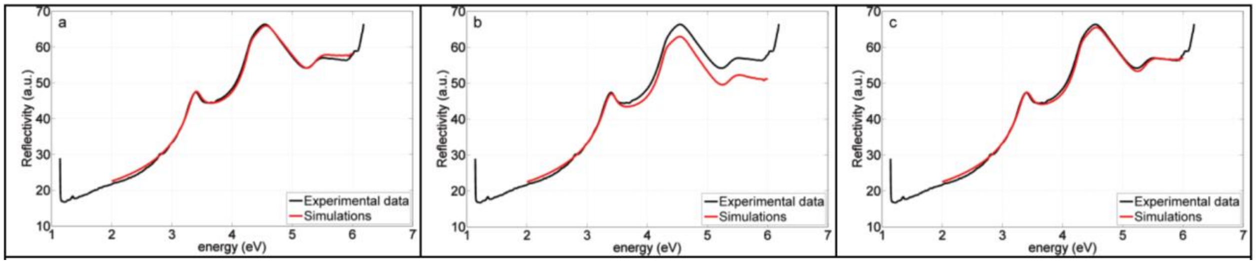

Figure S3: Comparison between the experimental (black curve) and the simulated (red curve) reflectivity spectrum of the substrate. The incident radiation is p-polarized, with $\Theta = 45^o$. (a) A substrate of Si is considered; (b) A layer, 2nm-thick, of SiO over a substrate of Si is considered; (c) A layer, 2nm-thick, of SiO_2 over a substrate of Si is considered. Experimental dielectric function data for Si, SiO and SiO_2 taken from [2]. Comparing the various panels, it is apparent that including an oxide Si layer does not improve the theoretical description of the experimental optical behavior. As such, a pure Si substrate has been considered in the simulations of the NP-containing systems.

References

[1] D'Addato, S.; Gragnaniello, L.; Valeri, S.; Rota, A.; di Bona, A.; Spizzo, F.; Panozaqi, T.; Schifano, S. F. J. *Appl. Phys.* **2010**, *107,* 104318.

[2] Palik, E. D. *Handbook of Optical Constants of Solids;* Academic Press: San Diego, USA, 1998.
